# A Lightweight End-to-End Framework for Real-Time Vehicle-Ejected Debris Detection on Edge Devices

**DOI:** 10.3390/s26144386

**Published:** 2026-07-10

**Authors:** Yichun Xu, Ning Chen, Haocheng Wen, Jianjun Zhuang

**Affiliations:** School of Electronic and Information Engineering, Nanjing University of Information Science and Technology, Nanjing 210044, China; 202383270446@nuist.edu.cn (Y.X.); 202383270383@nuist.edu.cn (N.C.); 202483270272@nuist.edu.cn (H.W.)

**Keywords:** vehicle-ejected debris detection, intelligent traffic enforcement, small-object detection, MobileNetV4-YOLOv8m, channel alignment, INT8 quantization, RDK X5 edge deployment

## Abstract

Vehicle-ejected debris detection is a practical but insufficiently studied problem in intelligent traffic enforcement. Unlike static road litter, objects thrown from moving vehicles are usually small, irregular, transient, and easily confused with road textures, shadows, lane markings, and light reflections. In current traffic management, such violations still rely heavily on manual video review or offline inspection, while task-specific datasets and edge-deployable detection solutions remain limited. To address this gap, this study constructs a vehicle-ejected debris dataset containing 4328 annotated image samples collected from real road scenarios. The dataset covers urban and suburban roads, daytime and nighttime illumination, near-range and distant small-object cases, and hard negative samples. To meet the coupled requirements of vehicle-mounted small-object detection and edge-side INT8 deployment, this study develops a hardware-aware lightweight detection framework based on YOLOv8m. The original CSPDarknet backbone is replaced with the convolutional variant of MobileNetV4 to reduce feature-extraction cost, while a scale-specific Channel Alignment Module is inserted between the heterogeneous MobileNetV4 backbone and the YOLOv8m PANet neck to preserve multi-scale feature compatibility. The alignment module uses only BPU-friendly convolution, batch normalization, and activation operations, thereby avoiding deployment-unfriendly operators while maintaining compatibility with INT8 quantization and edge acceleration. The trained FP32 model is quantized to INT8 and deployed on the RDK X5 BPU using the Horizon OpenExplorer toolkit. Experimental results and repeated-seed validation show that the proposed model achieves a consistent accuracy–efficiency advantage on the constructed dataset. In a representative run, the proposed model obtains 93.1% mAP_50_, while reducing the number of parameters from 25.9 M to 13.1 M and GFLOPs from 78.9 to 39.6 compared with the YOLOv8m baseline. After INT8 deployment, the model reaches 112.6 FPS on the RDK X5 platform with only a minor accuracy decrease. These results indicate that the proposed framework can serve as a practical edge-deployable perception module for real-time vehicle-ejected debris monitoring under vehicle-mounted traffic-enforcement scenarios. It should be noted that this work focuses on single-frame debris detection, while event-level ejection verification, temporal consistency analysis, offending-vehicle attribution, and enforcement decision-making remain beyond the scope of this study.

## 1. Introduction

Vehicles ejecting trash onto roadways is a recurring violation in urban traffic management. The ejected objects—plastic bottles, food wrappers, cigarette butts, and other small debris—create direct safety hazards for following vehicles and motorcyclists, contribute to roadside pollution, and clog drainage systems during rain [1]. Enforcement is difficult because the offending vehicle is moving, the object is small, and the action itself lasts only a fraction of a second. Most traffic agencies currently rely on roadside cameras with manual video review, which is labor-intensive and rarely catches violations in real time. Automating this perception stage on in-vehicle edge devices would allow potential debris-related events to be flagged and recorded in real time, without requiring constant human attention. Recent advancements in lightweight deep learning models, particularly YOLO-based architectures optimized for edge deployment, offer a promising solution for real-time, high-precision detection of such small and fast-moving waste targets [2,3,4].

Early work on road-debris detection followed classical computer vision pipelines: background subtraction, color thresholding, and hand-crafted shape or texture descriptors [5,6]. These methods perform reasonably under fixed-camera setups with clean backgrounds, but they degrade quickly when the camera itself moves, when lighting changes, or when the target shares texture with the road surface. Deep-learning-based detectors have largely replaced these approaches. Two-stage models such as Faster R-CNN [7] achieve strong accuracy but incur higher inference latency, while single-stage models from the YOLO family [8,9] trade a small accuracy margin for substantially faster predictions. For applications that must run in real time on embedded hardware, single-stage detectors are now the default choice.

Several recent works in the YOLO family have targeted small-object detection or lightweight deployment, both of which are directly relevant to vehicle-ejected debris. Liu et al. [10] integrated dynamic snake convolution and a deformable attention mechanism into YOLOv11n for multi-target detection in river and lake environments, reporting an mAP@0.5 of 83.9%. Gao et al. [11] replaced the C3K2 modules of YOLO11 with ShuffleNetV1 units and added SPD-Conv to preserve fine-grained features for forest fire detection, cutting parameters by 22.5% with a 0.3-point gain in mAP. For litter detection specifically, the pLitterStreet dataset [1] provides a benchmark for static roadside litter collected from vehicle-mounted cameras, and the RoLID-11K dataset [12] extends this to a larger collection of accumulated roadside objects. None of these works, however, addresses vehicle-ejected debris as a distinct task. The existing litter datasets focus on accumulated, weathered objects already settled on the ground, whose appearance and context differ markedly from objects in mid-flight or immediately after impact. Methods designed for those datasets do not necessarily transfer to the ejected-debris case, where the target is typically smaller, more irregular, and more often co-located with hard negatives such as fallen leaves or surface reflections. Recent studies on visual prompting and cooperative perception also provide useful references for road-scene understanding. For example, VRP-SAM introduces visual reference prompts into the Segment Anything framework to improve reference-guided segmentation, and visual in-context learning explores how reference examples can enhance visual recognition and adaptation [13]. CoopTrack further investigates end-to-end cooperative sequential perception for dynamic scenes [14]. These methods are relevant because they highlight the importance of reference information, temporal consistency, and cooperative perception in complex visual environments. However, they are not directly designed for lightweight single-frame debris detection on vehicle-mounted edge devices. In contrast, this study focuses on a compact detector that can be quantized to INT8 and deployed on the RDK X5 BPU for real-time vehicle-mounted debris perception. Transformer-based object detectors represent another important direction in recent object detection research. DETR formulates object detection as an end-to-end set prediction problem and introduces transformer encoder-decoder attention to model global object relationships [15]. Subsequent studies, such as Deformable DETR, Conditional DETR, DAB-DETR, DN-DETR, and DINO, further improve convergence speed, query formulation, multi-scale feature utilization, and detection accuracy [16]. Recent real-time transformer detectors, such as RT-DETR, also demonstrate the potential of transformer-based detection under real-time constraints [17]. In addition, recent work on rethinking the multi-scale feature hierarchy in object detection transformers provides useful insights into how feature hierarchy design affects detection performance [15]. These methods show strong global modeling capability and provide valuable references for multi-scale representation learning. However, they usually rely on attention-based feature interaction, transformer decoding, and more complex memory access patterns, which may increase deployment difficulty on resource-constrained INT8 edge accelerators. In contrast, this work focuses on a lightweight CNN-based detector for vehicle-mounted edge deployment. The proposed channel alignment strategy does not redesign the global feature hierarchy as in transformer-based detectors, but instead provides a simple and hardware-friendly bridge between the MobileNetV4 backbone and the YOLOv8m PANet neck to preserve multi-scale feature compatibility under INT8 deployment constraints.

Beyond task definition and related perception paradigms, a second gap concerns deployment. Most reported lightweight YOLO variants are evaluated on GPU benchmarks rather than on the embedded accelerators they are meant to serve, and quantization is often treated as an afterthought rather than as a co-design factor. For traffic-enforcement applications, however, the model must run on a vehicle-mounted edge device within a constrained power budget. Backbone choice, channel dimensioning, and operator compatibility with the target accelerator all affect the final deployed performance, and these factors are tightly coupled.

Recent lightweight YOLO variants frequently employ mobile backbones like MobileNet, ShuffleNet, or GhostNet to minimize parameters and computational costs. While effective for general object detection and embedded inference, these approaches typically view backbone replacement merely as a model compression tactic. However, detecting vehicle-ejected debris presents unique challenges: targets are often small and irregular, backgrounds contain complex hard negatives from road surfaces, and the model requires INT8 quantization for deployment on vehicle-mounted BPUs. Consequently, this work goes beyond simple backbone substitution in YOLOv8m. Instead, we integrate the convolutional variant of MobileNetV4 into the standard YOLOv8m PANet neck via scale-specific channel alignment. This design simultaneously addresses small-object multi-scale feature fusion and ensures compatibility with INT8 deployment.

This paper addresses the above gaps from a task-oriented and deployment-oriented perspective. Unlike many MobileNet-based YOLO variants that mainly focus on reducing model size on general detection benchmarks [18], the proposed framework is designed for vehicle-mounted debris detection, where targets are typically small, irregular, transient, and easily confused with road surface interference. In this setting, simply replacing the YOLOv8m backbone with a lightweight network may reduce computation but can also disturb the multi-scale feature structure required by the PANet neck. Therefore, we propose a hardware-aware MobileNetV4-YOLOv8m integration strategy. The convolutional variant of MobileNetV4 [19] is adopted to reduce feature-extraction cost and improve INT8 operator compatibility, while a scale-specific Channel Alignment Module is used to bridge the channel mismatch between MobileNetV4 and the YOLOv8m PANet neck. The module is intentionally kept lightweight and convolution-based so that it can preserve multi-scale feature compatibility without introducing attention-based or transformer-style operators that may be less suitable for BPU deployment [20]. The trained FP32 model is then quantized to INT8 and deployed on the RDK X5 BPU through the Horizon OpenExplorer toolkit, leveraging post-training quantization techniques that are proven essential for maximizing inference efficiency on resource-constrained neural processing units [21].

The main contributions of this work are summarized as follows:

- A rare vehicle-ejected debris dataset containing 4328 annotated image samples was collected from real vehicle-mounted road videos. The dataset covers different illumination conditions, road types, debris appearances, and typical road-surface interference. Hard negative samples, including fallen leaves, water reflections, and lane-marking fragments, were retained to improve the discriminative capability of the detector.
- A deployment-oriented MobileNetV4-YOLOv8m integration framework is designed for vehicle-mounted edge sensing. Different from conventional backbone-replacement strategies that mainly pursue model compression, the proposed framework jointly considers small-object feature preservation, heterogeneous feature-channel compatibility, and INT8-friendly BPU deployment. A scale-specific Channel Alignment Module is introduced to project the P3, P4, and P5 outputs of MobileNetV4 into the channel dimensions required by the YOLOv8m PANet neck, enabling the original neck and decoupled detection head to be retained with minimal additional computational cost.
- A complete edge deployment pipeline is implemented on the RDK X5 platform. The trained FP32 model is exported to ONNX, quantized to INT8, calibrated, and compiled into a BPU-executable model using the Horizon OpenExplorer toolkit. Experimental results show that the deployed model achieves 112.6 FPS on the RDK X5 BPU with only a small accuracy drop, demonstrating its feasibility for real-time vehicle-mounted traffic enforcement.

The remainder of this paper is organized as follows. Section 2 describes the proposed network architecture, including the MobileNetV4 backbone, the Channel Alignment Module, and the INT8 deployment pipeline. Section 3 presents the dataset construction, experimental setup, ablation study, comparison with representative detectors, and qualitative visualization. Section 4 concludes the paper and outlines limitations and future work.

## 2. Method

### 2.1. YOLOv8 Network Architecture

YOLOv8, released by Ultralytics in 2023, represents a significant advancement in single-stage object detection by integrating architectural improvements over its predecessors. The model has demonstrated state-of-the-art performance across numerous detection benchmarks and has been widely adopted in real-time vision tasks [22]. When applied to vehicle-ejected debris detection, YOLOv8’s anchor-free design, decoupled detection head, and distribution focal loss (DFL) collectively provide robust capability for localizing irregular small targets that frequently appear in dashcam imagery. Furthermore, YOLOv8 demonstrates favorable inference latency compared with two-stage detectors such as Faster R-CNN [7] and Transformer-based detectors such as RT-DETR [23]. Consequently, this study adopts YOLOv8m as the baseline model. Nevertheless, with the growing demand for deploying detection algorithms on resource-constrained edge platforms such as the RDK X5 for in-vehicle traffic enforcement, YOLOv8m requires further optimization in terms of lightweight design while maintaining its detection accuracy on extreme small-object scenarios.

**Figure 1:**
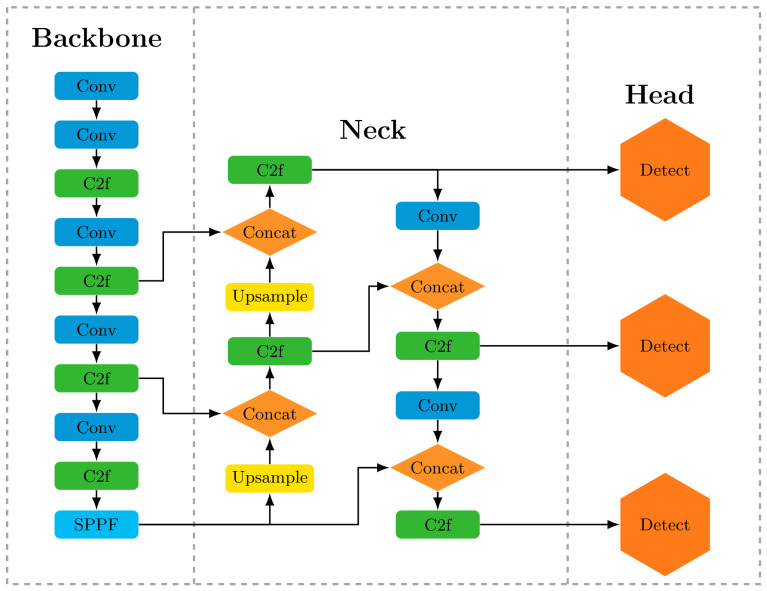
YOLOv8m baseline architecture.

The architecture of YOLOv8 comprises three core components: a CSPDarknet backbone, a Path Aggregation Network (PANet) neck, and a decoupled detection head. The CSPDarknet backbone employs Cross Stage Partial connections through C2f modules, which split feature maps into two branches and process them through a series of bottleneck blocks. This design reduces computational redundancy while maintaining strong feature representation capability [24]. The neck network integrates the PANet structure that performs bidirectional feature aggregation through a top-down semantic pathway and a bottom-up spatial pathway, enabling multi-scale feature fusion essential for detecting objects of varying sizes [25]. The detection head adopts a decoupled architecture, separating classification and bounding box regression into two parallel branches, which has been demonstrated to improve both convergence speed and detection accuracy over coupled designs [26,27]. The overall architecture of the YOLOv8m model is illustrated in Figure 1.

When YOLOv8m is applied to vehicle-ejected debris detection, the C2f modules in the backbone provide robust gradient flow through residual connections, mitigating gradient vanishing in deep layers and ensuring the network’s capability to extract discriminative features from small thrown objects. The PANet neck enables fine-grained spatial information to be propagated to deeper semantic layers, which is critical given that 67% of annotations in the constructed dataset fall in the small-object range. Nevertheless, despite these architectural advantages, the CSPDarknet backbone of YOLOv8m contains approximately 25.9 M parameters and incurs 78.9 GFLOPs of computational overhead at 640×640 input resolution. This computational footprint substantially exceeds the practical envelope of the RDK X5 BPU, which provides 10 TOPS of INT8 throughput. Furthermore, when conventional INT8 quantization is applied to the CSPDarknet backbone, the heterogeneous distribution of activation magnitudes across CSP branches induces non-trivial precision loss, exacerbating the trade-off between lightweighting and detection accuracy.

In summary, three coupled limitations of the baseline YOLOv8m prevent direct edge deployment: (1) excessive parameter count and computational overhead, (2) limited operator compatibility with INT8 BPU pipelines, and (3) degraded quantization stability of CSP-based feature representations. The following subsections describe architectural changes that address each of these issues.

### 2.2. Improvements to YOLOv8m

This study proposes a modified network architecture based on YOLOv8m that preserves detection accuracy while simultaneously enabling lightweight deployment on edge computing platforms. The modifications focus on three components: replacing the CSPDarknet backbone with MobileNetV4 (Section 2.2.1), introducing a Channel Alignment Module to bridge the channel mismatch (Section 2.2.2), and configuring an INT8 quantization pipeline for BPU deployment (Section 2.2.5).

From an edge-deployment perspective, the proposed architecture is designed according to three principles: reducing arithmetic complexity, maintaining multi-scale feature compatibility, and improving operator friendliness for INT8 acceleration. First, the original CSPDarknet backbone in YOLOv8m contains a relatively large number of standard convolutional and C2f operations, which leads to high parameter count and computational cost. For an input feature map with spatial size H×W, input channels $C_{\mathrm{in}}$, output channels $C_{\mathrm{out}}$, and kernel size *k*, the computational cost of a standard convolution can be approximated as

(1) $$C_{\mathrm{std}}=HWk^{2}C_{\mathrm{in}}C_{\mathrm{out}}.$$

In contrast, the depthwise-separable convolution used in MobileNet-style architectures decomposes this operation into a depthwise convolution and a pointwise convolution:

(2) $$C_{\mathrm{dw}+\mathrm{pw}}=HWk^{2}C_{\mathrm{in}}+HWC_{\mathrm{in}}C_{\mathrm{out}}.$$

Since $k^{2}C_{\mathrm{in}}+C_{\mathrm{in}}C_{\mathrm{out}}\ll k^{2}C_{\mathrm{in}}C_{\mathrm{out}}$ in most convolutional layers, this decomposition substantially reduces computation while preserving local spatial feature extraction. Therefore, replacing the original backbone with MobileNetV4 can reduce the computational burden of feature extraction, which is critical for deployment on the RDK X5 BPU.

Second, lightweighting the backbone alone is insufficient because the output channel dimensions of MobileNetV4 do not naturally match the input dimensions required by the YOLOv8m neck. Directly feeding mismatched or weakly adapted features into the PANet neck may damage multi-scale feature fusion, especially for small debris targets. The Channel Alignment Module is therefore introduced as a learnable projection layer. Its 1×1 convolution adjusts the channel dimensions at each scale without changing spatial resolution:

(3) $$F_{i}^{\mathrm{aligned}}=\delta \left(\mathrm{BN}\left(W_{i}^{1\times 1}*F_{i}^{\mathrm{MNv}4}\right)\right),\;i\in \left\{3,4,5\right\}.$$

This design allows the MobileNetV4 backbone to be connected to the original YOLOv8m neck while preserving the multi-scale detection structure. In this sense, the CAM does not simply increase or reduce channels; it acts as a lightweight feature adapter between two heterogeneous architectures.

Third, the proposed combination is also motivated by hardware compatibility. The RDK X5 BPU is optimized for regular convolutional operators and INT8 inference. Compared with attention-based or highly customized operators, convolution, depthwise convolution, pointwise convolution, batch normalization, and activation operations are more suitable for static graph conversion, quantization calibration, and BPU compilation. Therefore, the proposed MobileNetV4-YOLOv8m architecture is not only lightweight in terms of parameter count and GFLOPs, but also more compatible with the target edge-deployment pipeline.

#### 2.2.1. Replacing the CSPDarknet Backbone with MobileNetV4

To address the challenge of excessive parameter count and computational overhead introduced by the CSPDarknet backbone in YOLOv8m-based detection models [28], limitations that hinder real-time deployment on the RDK X5 BPU [29], this study replaces the entire CSPDarknet backbone of the baseline model with the lightweight MobileNetV4 architecture [30].

MobileNetV4, introduced by Google in 2024, represents the latest generation of mobile-optimized convolutional networks. Architected around the Universal Inverted Bottleneck (UIB) and Neural Architecture Search (NAS)-driven design principles, MobileNetV4 achieves favorable accuracy–efficiency trade-offs across diverse hardware platforms ranging from mobile CPUs to dedicated AI accelerators. Compared with earlier lightweight CNN families, MobileNetV4 provides a more recent hardware-aware design for mobile and edge deployment scenarios, making it suitable for resource-constrained object detection tasks [31].

The core advantage of MobileNetV4 arises from the synergistic integration of the Universal Inverted Bottleneck and hardware-friendly depthwise-separable convolutions. This design paradigm not only enables substantial model lightweighting but also preserves sufficient representational capacity for fine-grained feature extraction. The specific functionalities and underlying mechanisms of these core components are elaborated as follows.

(1) Universal Inverted Bottleneck (UIB)

The Universal Inverted Bottleneck unifies multiple convolutional structures within a single, hardware-aware framework. A UIB block consists of two pointwise convolutions and two optional depthwise convolutions, whose activation is controlled by binary switches. Formally, the UIB operation is expressed as:

(4) $$\mathrm{UIB}\left(x\right)={\mathrm{PWConv}}_{2}\left({\mathrm{DWConv}}_{2}^{\alpha }\left({\mathrm{PWConv}}_{1}\left({\mathrm{DWConv}}_{1}^{\beta }\left(x\right)\right)\right)\right)+x.$$

where α,β∈{0,1} are switches controlling the presence of the two depthwise convolutions. Through different combinations of α and β, the UIB block can be configured into four functionally distinct variants, as summarized below:

(5) $$\mathrm{Variant}=\left\{\begin{array}{cc} \mathrm{ConvNeXt-like}, & \alpha =1,\;\beta =0,\\ \mathrm{Inverted}\;\mathrm{Residual}, & \alpha =0,\;\beta =1,\\ \mathrm{ExtraDW}, & \alpha =1,\;\beta =1,\\ \mathrm{FFN}, & \alpha =0,\;\beta =0. \end{array}\right.$$

**Figure 2:**
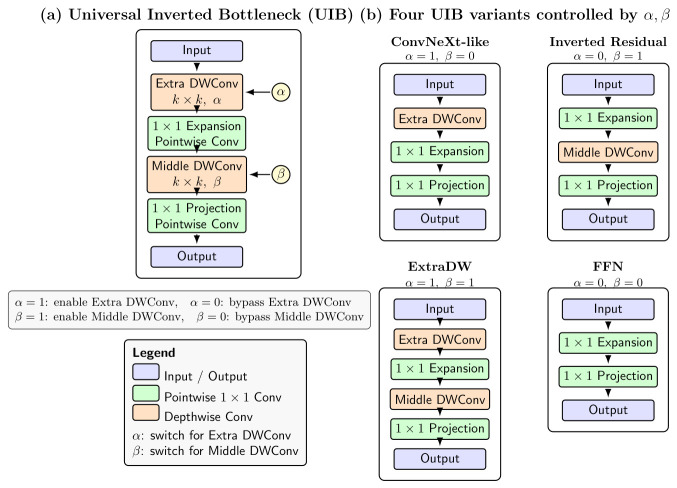
UIB block structure and four variants controlled by α and β.

This flexibility allows NAS-based optimization to select the most suitable variant for each stage of the network based on the target hardware. The structure of the UIB block and its four variants is illustrated in Figure 2.

(2) Conv Variant Selection for Edge Deployment

MobileNetV4 provides two architectural families: the conv variant comprising purely convolutional layers, and the hybrid variant incorporating Mobile Multi-Query Attention (Mobile MQA) modules. While the hybrid variant achieves marginally higher accuracy on classification benchmarks, its attention modules introduce two practical concerns for edge deployment: first, the softmax operation within Mobile MQA exhibits high sensitivity near zero, causing substantial precision degradation under INT8 quantization [32]; second, certain attention-related operators may not be natively supported by the RDK X5 BPU [33] instruction set, requiring fallback to CPU execution and inducing latency penalties. To circumvent these issues, this study adopts the conv variant (MobileNetV4-Conv-Medium), whose purely convolutional design guarantees full operator compatibility with the BPU and minimizes quantization-induced accuracy loss [34]. The detailed stage configuration of MobileNetV4-conv-medium is presented in Table 1.

Replacing CSPDarknet with MobileNetV4 thus addresses three of the limitations identified in Section 2: it reduces parameter count and computational overhead, improves quantization stability through more uniform activation distributions across depthwise-separable layers, and enables deployment on the RDK X5 BPU within its INT8 instruction set.

#### 2.2.2. Channel Alignment Module

While replacing the backbone of YOLOv8m with MobileNetV4 yields substantial efficiency gains, a critical integration challenge arises from the architectural inconsistency between the two networks [35]. As shown in Table 1, MobileNetV4-conv-medium produces feature maps with channels of (80, 160, 960) at the P3, P4, and P5 stages, respectively, whereas the YOLOv8m PANet neck expects input channels of (192, 384, 768) at the corresponding scales. This discrepancy extends beyond mere tensor dimensionality; it also affects the channel distribution and semantic density of multi-scale features, which are important for the top-down and bottom-up feature aggregation in PANet. If MobileNetV4 features are directly connected to the YOLOv8m neck, or only adapted through naive padding or cropping operations, the multi-scale fusion capability of the original neck may be weakened, especially for small debris targets that rely heavily on high-resolution shallow features [36].

To address this issue, this study introduces a scale-specific Channel Alignment Module (CAM) positioned between the MobileNetV4 backbone and the YOLOv8m neck. Different from a single shared channel adapter, CAM contains three independent alignment branches for P3, P4, and P5. Each branch consists of a pointwise (1×1) convolution followed by batch normalization and SiLU activation [20]. The purpose of this design is not only to match the channel dimensions, but also to provide a lightweight learnable projection for adapting MobileNetV4 features to the feature space expected by the YOLOv8m PANet neck. Formally, the alignment operation is expressed as:

(6) $$F_{i}^{\mathrm{aligned}}=\delta \left(\mathrm{BN}\left(W_{i}^{1\times 1}*F_{i}^{\mathrm{MNv}4}\right)\right),\;i\in 3,4,5$$

where $W_{i}^{1\times 1}$ denotes the 1×1 convolution kernel at the *i*-th scale, δ(·) represents the SiLU activation function, and BN(·) denotes batch normalization.

**Figure 3:**
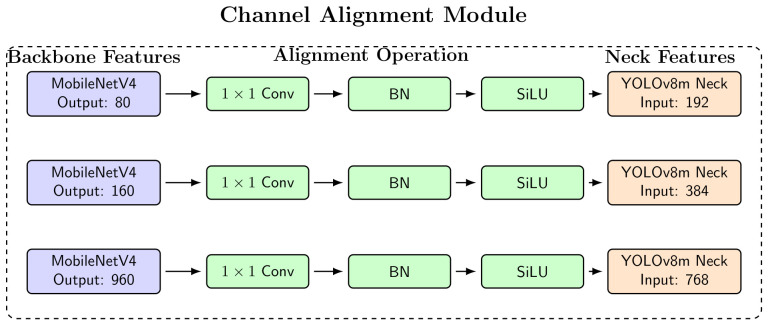
Channel Alignment Module for mapping MobileNetV4 features to YOLOv8m neck inputs.

The CAM performs scale-dependent channel transformation. At P3 and P4, the channels are expanded from 80 to 192 and from 160 to 384, respectively, so that shallow and middle-level MobileNetV4 features can provide sufficient channel capacity for the YOLOv8m PANet. This is particularly important for vehicle-ejected debris detection, because the P3 feature map retains relatively high spatial resolution and contributes strongly to small-object localization. At P5, the channels are compressed from 960 to 768, which reduces the computational burden of subsequent neck operations while retaining high-level semantic information. Therefore, CAM performs not merely dimensional matching, but scale-dependent feature adaptation for multi-scale detection. The detailed structure of the CAM is illustrated in Figure 3.

It should be emphasized that the contribution of CAM does not lie in the use of 1×1 convolution alone, which is a standard operation. Instead, CAM is designed as a scale-specific and deployment-oriented adapter for integrating two heterogeneous architectures under the constraints of vehicle-mounted small-object detection and INT8 BPU inference. More complex feature fusion modules, such as attention-based fusion, transformer-based interaction, or deformable feature adaptation, may provide stronger feature interaction in general detection tasks. However, they often introduce additional memory access, quantization sensitivity, or deployment-unfriendly operators, which may reduce the stability of INT8 quantization and BPU compilation on the target edge platform. Therefore, they were not adopted as the main adaptation module in this work. By using only convolution, batch normalization, and activation operations that are well supported by the Horizon OpenExplorer toolchain and the RDK X5 BPU, CAM provides a lightweight and hardware-compatible feature adaptation bridge between MobileNetV4 and the YOLOv8m PANet neck. In this sense, CAM is not intended to maximize feature fusion complexity, but to achieve a practical balance among feature adaptation capability, computational overhead, quantization compatibility, and edge-deployment feasibility. From the perspective of recent transformer-based detectors, the proposed CAM follows a different design objective. DETR-style methods usually improve detection by redesigning attention-based feature interaction, object queries, or multi-scale feature hierarchy within a transformer encoder-decoder framework. In contrast, CAM does not attempt to reconstruct the global feature hierarchy or introduce transformer decoding. Instead, it performs scale-specific channel projection at P3, P4, and P5, so that the heterogeneous MobileNetV4 backbone can be effectively connected to the YOLOv8m PANet neck while retaining convolution-only, INT8-friendly deployment compatibility.

#### 2.2.3. Overall Architecture of the Proposed Model

**Figure 4:**
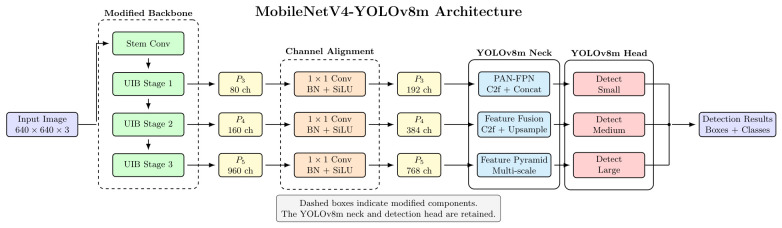
Architecture of the proposed MobileNetV4-YOLOv8m model.

Building on the aforementioned modifications, the network architecture of the proposed MobileNetV4-YOLOv8m model is illustrated in Figure 4. The MobileNetV4-conv-medium backbone replaces the CSPDarknet of the baseline, producing multi-scale feature maps at P3, P4, and P5. These features are then transformed by the Channel Alignment Module to match the input dimensions expected by the YOLOv8m PANet neck. The PANet performs bidirectional feature aggregation through top-down and bottom-up pathways, and the aligned and fused features are passed to the original YOLOv8m decoupled detection head for final prediction. The enhanced model preserves the complete neck and head architecture of the baseline YOLOv8m to maintain inference efficiency, while achieving simultaneous improvements in model lightweighting and quantization-friendliness through structural optimization of the backbone.

As shown in Figure 4, the MobileNetV4 backbone and the Channel Alignment Module are integrated into the improved YOLOv8m framework to achieve a better balance between lightweight design and detection accuracy for vehicle-ejected debris detection. The effectiveness of the integrated modules is verified through the experiments presented in Section 3. Overall, the proposed architecture follows an edge-oriented co-design strategy: MobileNetV4 reduces the feature extraction cost, CAM restores the channel compatibility required by the YOLOv8m neck, and the retained PANet and decoupled head preserve the original multi-scale detection capability. This combination enables the detector to maintain accuracy for small debris targets while improving compatibility with INT8-based BPU deployment.

#### 2.2.4. Loss Function

The proposed model retains the original YOLOv8 loss formulation, which combines three complementary objectives to jointly optimize bounding box localization, class probability prediction, and distribution-based regression refinement. The total loss is expressed as:

(7) $$L_{\mathrm{total}}=\lambda_{\mathrm{box}}L_{\mathrm{CIoU}}+\lambda_{\mathrm{cls}}L_{\mathrm{BCE}}+\lambda_{\mathrm{dfl}}L_{\mathrm{DFL}}$$

The Complete Intersection over Union (CIoU) loss measures bounding box localization quality by jointly considering overlap area, center-point distance, and aspect ratio consistency:

(8) $$L_{\mathrm{CIoU}}=1-\mathrm{IoU}\left(b,b^{gt}\right)+\frac{\rho^{2}\left(b,b^{gt}\right)}{c^{2}}+\alpha v.$$

where *b* and $b^{gt}$ denote the predicted and ground truth bounding box centers, ρ(·) is the Euclidean distance, *c* is the diagonal length of the smallest enclosing box covering both boxes, *v* quantifies aspect ratio consistency, and α=v/((1−IoU)+v) is a positive trade-off coefficient that increases the penalty when both IoU is low and the aspect ratio is inconsistent.

The Binary Cross-Entropy (BCE) loss handles class probability prediction:

(9) $$L_{\mathrm{BCE}}=-\frac{1}{N}\sum_{i=1}^{N}\left[y_{i}\log\left({\hat{y}}_{i}\right)+\left(1-y_{i}\right)\log\left(1-{\hat{y}}_{i}\right)\right]$$

The Distribution Focal Loss (DFL) reformulates bounding box regression as a probability distribution prediction problem:

(10) $$L_{\mathrm{DFL}}=-\left(\left(y_{i+1}-y\right)\log\left(S_{i}\right)+\left(y-y_{i}\right)\log\left(S_{i+1}\right)\right)$$

DFL encourages the network to focus on values around the ground truth, which is particularly beneficial in our setting, where 67% of annotations fall in the small-object range and are sensitive to even a few-pixel localization error. The loss weights $\lambda_{\mathrm{box}}=7.5$, $\lambda_{\mathrm{cls}}=0.5$, and $\lambda_{\mathrm{dfl}}=1.5$ follow the default YOLOv8 settings and are kept consistent across all experiments to ensure fair comparison.

#### 2.2.5. INT8 Quantization for BPU Deployment

To enable real-time inference on the RDK X5 BPU, which provides 10 TOPS of INT8 computational throughput at approximately 5W power consumption, the trained FP32 model is converted to INT8 precision through post-training quantization (PTQ). The quantization pipeline comprises four stages: (1) ONNX export from the PyTorch model, (2) operator compatibility verification against the BPU instruction set, (3) per-layer scale factor calibration using a representative subset of 200 training images, and (4) compilation to a BPU-executable binary using the Horizon OpenExplorer toolkit.

**Figure 5:**
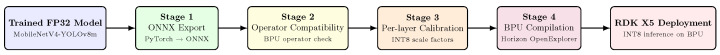
INT8 quantization and BPU deployment pipeline. The pipeline comprises four stages: ONNX export, operator compatibility verification, per-layer calibration, and BPU compilation.

Symmetric quantization is adopted for both weights and activations, mapping FP32 tensors to the INT8 range [−128,127]:

(11) $$x_{\mathrm{int}8}=\mathrm{clip}\left(\mathrm{round}\left(\frac{x_{\mathrm{fp}32}}{s}\right),-128,127\right),\;s=\frac{\max(|x_{\mathrm{calib}}|)}{127}.$$

where *s* is the per-layer scale factor determined from calibration data. The quantized matrix multiplication is then computed in the INT8 domain:

(12) $$y\approx s_{x}\cdot s_{W}\cdot \left(x_{\mathrm{int}8}\cdot W_{\mathrm{int}8}\right).$$

where $s_{x}$ and $s_{W}$ denote the scale factors of activations and weights respectively. The choice of MobileNetV4’s conv variant in Section 2.2.1 is motivated in part by this stage: by avoiding attention-based operators whose softmax activations are known to be sensitive to INT8 quantization, the network is expected to incur smaller precision loss during quantization. The actual quantization-induced accuracy drop is measured and reported in Section 3. The detailed quantization deployment pipeline is illustrated in Figure 5.

## 3. Experiments and Results

### 3.1. Dataset Construction

An experimental dataset was prepared to evaluate the proposed model under vehicle-mounted road-scene conditions. The dataset was built from 42 real road videos collected by vehicle-mounted cameras (Yahboom Technology Co., Ltd., Shenzhen, China) and supplemented with data augmentation to improve scene diversity. The source videos were recorded under real driving conditions, with a total acquisition duration of approximately 90 min. The original videos were captured from a front-view vehicle-mounted camera at a resolution of 1920 × 1080 and a frame rate of 30 FPS. The recording scenes include urban roads, suburban roads, intersections, straight road segments, and low-light road environments. The original frames include road scenes containing vehicle-ejected debris as well as visually similar interference objects, such as fallen leaves, lane-marking fragments, water reflections, tire marks, and small roadside objects. The data acquisition and annotation settings are summarized in Table 2.

After frame extraction, manual filtering, annotation, and data augmentation, a total of 4328 image samples were prepared for model training and evaluation. The target objects include plastic bags, paper boxes, beverage bottles, food wrappers, and other irregular debris visible on or near the road surface. Since the main objective of this work is to identify debris-ejection events for traffic enforcement rather than to perform fine-grained waste classification, all debris types were merged into a single class, denoted as “throwing debris”. Although this single-class setting is sufficient for detecting violation-related debris events, different debris appearances may indeed lead to different levels of detection difficulty. For example, deformable objects such as plastic bags or food wrappers may be more easily confused with road reflections or lane-marking fragments, while rigid objects such as bottles and boxes usually have clearer boundaries. Since fine-grained debris-type annotation requires substantially more data collection and labeling effort, category-level analysis is left as an important direction for the next stage of this work, where a larger and more diverse dataset will be constructed to support debris-type-specific evaluation.

The annotation protocol was defined as follows. All visible debris objects on or near the road surface were annotated using tight bounding boxes in YOLO format. For partially occluded objects, only the visible region was annotated. Objects with severe motion blur, extreme occlusion, or unclear boundaries were excluded from the final dataset to avoid ambiguous labels. Hard negative samples, such as fallen leaves, lane-marking fragments, water reflections, tire marks, and small roadside objects, were retained without debris labels to encourage the detector to distinguish true ejected debris from visually similar road-surface interference.

To ensure annotation quality, the initial annotations were manually checked after labeling. Images with missing labels, inaccurate bounding boxes, or ambiguous object boundaries were corrected through a secondary review. In addition, a subset of the annotated images was randomly inspected to further reduce labeling errors. This quality-control process was used to improve annotation consistency before the dataset was divided into training, validation, and test subsets.

To improve robustness under different sensing conditions, augmentation operations such as brightness adjustment, contrast variation, scaling, horizontal flipping, slight blur simulation, and noise perturbation were applied. These operations were used to simulate common variations in vehicle-mounted perception, including illumination changes, camera motion, and low-contrast road backgrounds.

The dataset was split into training, validation, and test sets with a 7:2:1 ratio. To mitigate information leakage from highly similar consecutive frames, all samples from the same video segment were kept within the same subset. The split details are summarized in Table 3.

Due to privacy concerns involving vehicle-mounted road videos—such as license plates, geographic locations, surrounding vehicles, and pedestrians—the complete raw videos and the full dataset are not publicly available at this stage. However, the dataset, annotation files, and split information can be obtained from the corresponding author upon reasonable request for research purposes, subject to applicable privacy and data-use restrictions.

Since the scale distribution of targets has a direct effect on detector design, the bounding box area of annotated instances was further analyzed. Following the COCO definition, objects with an area smaller than $32^{2}$ pixels are categorized as small, those with an area between $32^{2}$ and $96^{2}$ pixels are categorized as medium, and those with an area larger than $96^{2}$ pixels are categorized as large. The results are reported in Table 4. Small objects account for the majority of annotated instances, indicating that vehicle-ejected debris detection is dominated by small-target localization under typical vehicle-mounted camera geometry.

### 3.2. Experimental Environment

All training and evaluation experiments were performed under identical hardware and software configurations to ensure a fair comparison. The codebase is built on the Ultralytics YOLOv8 framework with PyTorch (PyTorch v2.8.0 (Meta AI, Menlo Park, CA, USA)) as the backend. Input images are uniformly resized to 640×640, and identical data augmentation pipelines are applied across all compared models unless otherwise specified.

Edge deployment was carried out on the RDK X5 platform, which integrates a 10 TOPS INT8 BPU accelerator. The trained FP32 model was first exported to ONNX, then quantized and compiled into a BPU-executable model using the Horizon OpenExplorer toolkit. During edge-side testing, a 2-megapixel high-definition camera was used as the visual input device to capture road-scene video frames. The captured frames were resized to the same input resolution as the training and evaluation setting, namely 640×640, and were processed directly on the RDK X5 platform. The deployed INT8 model performed debris-ejection recognition on the BPU with a batch size of 1 to simulate real-time vehicle-mounted inference.

The reported INT8 model FPS was measured on the target RDK X5 platform after model loading and warm-up. Note that FP32 FPS values for comparison were obtained on a training workstation, whereas INT8 FPS was measured on the RDK X5 BPU. Thus, the INT8 deployment results evaluate the real-time feasibility of the proposed model on the target edge accelerator, rather than providing a direct cross-platform speed comparison with FP32 workstation results. The full experimental environment is detailed in Table 5.

**Figure 6:**
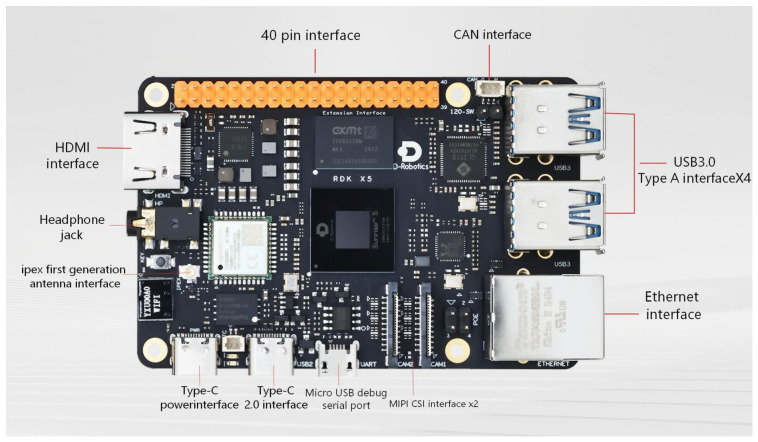
Hardware interface layout of the RDK X5 development board used for edge-side deployment. The platform provides camera, USB, Ethernet, CAN, Type-C power, and expansion interfaces for vehicle-mounted sensing and real-time inference.

For edge-side deployment, the RDK X5 development board was adopted as the target hardware platform. As shown in Figure 6, the board provides multiple peripheral interfaces, including USB 3.0 Type-A ports, an Ethernet interface, Type-C power input, MIPI CSI camera interfaces, a CAN interface, and a 40-pin expansion interface. These hardware interfaces support camera input, external communication, and real-time vehicle-mounted sensing, while the onboard BPU enables INT8 acceleration for the proposed lightweight detection model.

The training hyperparameters are summarized in Table 6. The baseline YOLOv8m and all variants of the proposed model were trained under identical settings to ensure that performance differences mainly resulted from architectural changes rather than hyperparameter tuning.

Loss weights followed the official YOLOv8 defaults and were not tuned, in order to isolate the effect of architectural changes from hyperparameter optimization. The MobileNetV4 backbone was initialized with ImageNet-pretrained weights from the timm library; the Channel Alignment Module and the YOLOv8m neck and head were trained from scratch.

For reproducibility, the dataset split files were fixed after the initial partition and were kept unchanged for all experiments. Unless otherwise specified, the representative training results were obtained using a fixed random seed of 42. To evaluate the robustness of the reported performance against training randomness, repeated experiments were additionally conducted for the YOLOv8m baseline and the proposed MobileNetV4-YOLOv8m model using five random seeds: 0, 1, 42, 2024, and 2026. In these repeated runs, the dataset split, input resolution, training epochs, batch size, optimizer, learning rate, data augmentation strategy, and loss weights were kept unchanged. Only the random seed controlling weight initialization, data shuffling, and stochastic augmentation was varied. During validation, all compared models followed the same non-maximum suppression setting, with an IoU threshold of 0.7 and the default validation confidence threshold of the Ultralytics framework. The results are reported as the mean and standard deviation over repeated runs.

### 3.3. Evaluation Metrics

Both accuracy and efficiency metrics were used to evaluate model performance. For accuracy, we report precision (*P*), recall (R), $mAP_{50}$, and $mAP_{50:95}$. For efficiency, we report parameter count, GFLOPs, and inference speed in frames per second (FPS).

Precision and recall are defined as:

(13) $$P=\frac{TP}{TP+FP},\;R=\frac{TP}{TP+FN}$$

where TP, FP, and FN denote true positives, false positives, and false negatives, respectively.

Average Precision (AP) is the area under the precision–recall curve, and mean Average Precision (mAP) is the mean of AP values across all categories:

(14) $$mAP=\frac{1}{N}\sum_{i=1}^{N}AP_{i}$$

where *N* is the number of categories. Since this work formulates the task as a single-class detection problem, N=1, and the AP for the single class is equivalent to mAP. We report $mAP_{50}$ (IoU threshold = 0.5) for overall detection quality and $mAP_{50:95}$ (averaged over IoU thresholds from 0.5 to 0.95 with a step of 0.05) for localization precision.

Frames per second is computed as:

(15) $$FPS=\frac{1}{T_{\mathrm{infer}}}$$

where $T_{\mathrm{infer}}$ is the average per-image inference time, measured over the test set under batch size 1 to reflect realistic deployment latency.

To evaluate whether the reported performance difference is robust to training randomness, repeated-seed experiments were further conducted. For these experiments, the dataset split, input resolution, training epochs, batch size, optimizer, learning rate, data augmentation strategy, and loss weights were kept unchanged, while only the random seed was varied. The random seed affects weight initialization, data shuffling, and stochastic data augmentation. For each model, the results are reported as the mean and standard deviation over repeated runs.

For a metric value $x_{j}$ obtained from the *j*-th run, the mean and standard deviation are calculated as:

(16) $$\bar{x}=\frac{1}{n}\sum_{j=1}^{n}x_{j},$$

(17) $$s=\sqrt{\frac{1}{n-1}\sum_{j=1}^{n}{\left(x_{j}-\bar{x}\right)}^{2}},$$

where *n* denotes the number of repeated runs. The 95% confidence interval of mAP50 is estimated using Student’s *t*-distribution:

(18) $$CI_{95\%}=\bar{x}\pm t_{0.975,n-1}\frac{s}{\sqrt{n}}.$$

This statistical reporting is used to assess the stability of the observed performance difference under different training seeds.

### 3.4. Ablation Study

To evaluate the contribution of each architectural component, an ablation study was first conducted by progressively modifying the YOLOv8m baseline. In addition to the component-wise ablation, three supplementary analyses were performed under the same experimental protocol. Specifically, repeated-seed experiments were conducted to assess the robustness of the observed performance difference to training randomness; different channel adaptation strategies were compared to examine the effectiveness of the proposed Channel Alignment Module (CAM); and several representative lightweight backbones were evaluated to justify the selection of MobileNetV4-conv-medium.

As shown in Table 7, replacing the original CSPDarknet backbone with MobileNetV4 substantially reduces the model complexity. The number of parameters decreases from 25.9 M to 12.8 M, while the inference speed increases from 42.3 FPS to 72.5 FPS. It should be noted that the “MobileNetV4 backbone + minimal neck-channel adjustment (no CAM)” setting does not mean that the MobileNetV4 features are directly connected to the original YOLOv8m neck without any dimensional modification. Since the output channels of MobileNetV4 at P3, P4, and P5 are different from the input channels expected by the original YOLOv8m PANet neck, this variant was implemented by only adjusting the input-channel dimensions of the corresponding neck layers to make the network runnable. No additional scale-specific Channel Alignment Module, batch normalization, or nonlinear activation was inserted between the backbone and the neck in this setting. Therefore, this variant serves as a minimal runnable baseline for evaluating whether the proposed CAM provides benefits beyond simple architectural compatibility.

Although this minimal adjustment improves computational efficiency, it also leads to a noticeable accuracy degradation, with $mAP_{50}$ decreasing from 92.5% to 90.1% and $mAP_{50:95}$ decreasing from 58.1% to 55.2%. This result suggests that simple channel compatibility alone is insufficient to preserve the multi-scale feature representation required by the PANet neck, thereby reducing feature-fusion and localization performance.

After introducing CAM, the proposed MobileNetV4-YOLOv8m model achieves 93.1% $mAP_{50}$ and 58.9% $mAP_{50:95}$ in the representative run, corresponding to improvements of 0.6 and 0.8 percentage points over the YOLOv8m baseline, respectively. Since the absolute improvement in $mAP_{50}$ is relatively moderate, repeated-seed experiments were further conducted to evaluate whether the observed performance difference remains stable under training randomness. The results are summarized in Table 8.

As shown in Table 8, the proposed MobileNetV4-YOLOv8m model maintains a consistent advantage over the YOLOv8m baseline across different random seeds. The mean $mAP_{50}$ increases from 92.50±0.16% to 93.08±0.15%, while the mean $mAP_{50:95}$ increases from 58.10±0.16% to 58.88±0.15%. The 95% confidence intervals of $mAP_{50}$ further indicate that the observed improvement is not attributable to a single favorable training run. More importantly, this accuracy level is achieved with a substantially lower computational burden, reducing the parameter count from 25.9 M to 13.1 M and the computational cost from 78.9 GFLOPs to 39.6 GFLOPs. Therefore, the advantage of the proposed framework should be interpreted primarily as an improved accuracy–efficiency trade-off rather than as a large absolute accuracy gain.

After INT8 quantization and deployment on the RDK X5 BPU, the proposed model reaches 112.6 FPS, with only a 0.9-point decrease in $mAP_{50}$ compared with its FP32 counterpart. This result indicates that the proposed architecture remains effective after quantization and is suitable for real-time edge inference.

To further examine the role of CAM, several channel adaptation strategies were compared, as summarized in Table 9. The channel padding/cropping strategy adjusts feature dimensions by zero-padding insufficient channels and truncating excessive channels. Although this operation introduces almost no additional parameters, it lacks learnable feature transformation and may discard useful information. Consequently, its improvement over the minimal channel matching setting is limited.

Compared with padding/cropping, the plain 1×1 convolution introduces a learnable projection between the MobileNetV4 backbone and the YOLOv8m neck, leading to improved detection performance. Nevertheless, its performance remains lower than that of the proposed CAM. Specifically, CAM improves $mAP_{50}$ from 91.8% to 93.1% and $mAP_{50:95}$ from 57.1% to 58.9%, with only a minor increase in parameters and computational cost. This improvement can be attributed to the scale-specific design of CAM, where independent alignment branches are applied to P3, P4, and P5 feature maps, followed by batch normalization and SiLU activation. Therefore, CAM serves not only as a channel-matching operation, but also as a lightweight nonlinear feature adapter between the MobileNetV4 backbone and the YOLOv8m PANet neck.

To further analyze the internal design of CAM, an additional ablation study was conducted by progressively enabling batch normalization, SiLU activation, scale-specific mapping, and different feature scales. The purpose of this experiment is to examine whether the performance gain of CAM comes only from the 1×1 convolution, or from the combined effect of normalization, nonlinear activation, and scale-specific feature adaptation. The results are summarized in Table 10.

As shown in Table 10, the plain 1×1 convolution achieves 91.8% $mAP_{50}$ and 57.1% $mAP_{50:95}$, indicating that a learnable channel projection is more effective than simple channel padding or cropping. However, its performance is still lower than that of the full CAM, suggesting that channel projection alone is insufficient to fully adapt the MobileNetV4 features to the YOLOv8m PANet neck. After adding batch normalization, $mAP_{50}$ increases from 91.8% to 92.2%, and $mAP_{50:95}$ increases from 57.1% to 57.6%. This improvement indicates that BN helps stabilize the channel responses before multi-scale feature fusion. When SiLU activation is further introduced, the performance improves to 92.6% $mAP_{50}$ and 58.0% $mAP_{50:95}$, showing that nonlinear feature transformation is also beneficial for feature adaptation.

The scale-related ablation further demonstrates the importance of scale-specific mapping. The non-scale-specific mapping strategy obtains 92.4% $mAP_{50}$ and 57.8% $mAP_{50:95}$, which is lower than the full scale-specific CAM. This result suggests that using the same mapping strategy for P3, P4, and P5 is not optimal, because different feature levels contain different spatial resolutions and semantic information. Among the single-scale variants, P3 alignment achieves the best performance, with 92.5% $mAP_{50}$ and 57.9% $mAP_{50:95}$, while P4-only and P5-only alignment obtain 92.0% and 91.9% $mAP_{50}$, respectively. This indicates that the high-resolution P3 feature map contributes more strongly to small debris localization, whereas P4 and P5 provide complementary middle-level and high-level semantic cues. By jointly aligning P3, P4, and P5 with independent branches, the full CAM achieves the best overall performance, reaching 93.1% $mAP_{50}$ and 58.9% $mAP_{50:95}$. Therefore, the proposed CAM is not simply a plain 1×1 convolution, but a scale-specific lightweight feature adaptation module that combines learnable projection, normalization, nonlinear activation, and multi-scale feature alignment.

From the perspective of intermediate feature distribution, using only minimal neck-channel adjustment mainly solves the tensor-size compatibility problem, but it does not sufficiently adapt the feature statistics and semantic density of different scales. This mismatch is particularly important for vehicle-ejected debris detection because small debris targets rely heavily on the high-resolution P3 feature map, while road textures, lane markings, shadows, and reflections may introduce strong background interference. The proposed CAM applies independent learnable projections to P3, P4, and P5, and uses batch normalization and SiLU activation to normalize and nonlinearly transform the feature responses before PANet fusion. As a result, the aligned features are more compatible with the feature space expected by the YOLOv8m neck, which helps preserve small-object localization cues while reducing the negative effect of mismatched multi-scale feature distributions. This explains why CAM outperforms direct channel matching, padding/cropping, and plain channel adaptation in Table 9.

Finally, different lightweight backbones were compared under the same YOLOv8m neck and decoupled detection head to justify the selection of MobileNetV4-conv-medium. For a fair comparison, all lightweight backbones were equipped with CAM to align their multi-scale output features with the PANet neck. The dataset split, input resolution, training configuration, and evaluation metrics were kept identical across all compared models.

As shown in Table 11, all lightweight backbones reduce the parameter count and improve inference speed compared with the original CSPDarknet-based YOLOv8m baseline. ShuffleNetV2 achieves the highest inference speed among the evaluated lightweight backbones, but its $mAP_{50}$ and $mAP_{50:95}$ are clearly lower, suggesting that excessive lightweighting may weaken the feature representation required for small and irregular debris targets. GhostNet provides a better balance between compactness and accuracy than ShuffleNetV2, while MobileNetV3-Large further improves detection accuracy.

Among the evaluated lightweight backbones, MobileNetV4-conv-medium obtains the highest $mAP_{50}$ of 93.1% and the highest $mAP_{50:95}$ of 58.9%, while reducing the parameter count by nearly half compared with the YOLOv8m baseline. Although it is not the fastest lightweight backbone, it provides the most favorable balance between detection accuracy and computational efficiency in this task. Based on these results, MobileNetV4-conv-medium is selected as the backbone of the proposed framework.

### 3.5. Comparison with Existing Methods

To further evaluate the effectiveness of the proposed method, we compared it with several representative object detection models, including a two-stage detector, conventional YOLO-series detectors, and recent lightweight real-time detectors. In addition to Faster R-CNN and YOLOv8 variants, YOLOv10s and YOLOv11s/m were included because they represent recent accuracy–efficiency-oriented detectors that are commonly considered for real-time and edge-side object detection. All models were trained and evaluated on the same dataset split with the same input resolution and comparable training settings where applicable. The comparison focuses on practical real-time detectors rather than exhaustively covering all possible object detection paradigms. The comparison results are summarized in Table 12.

As shown in Table 12, the proposed MobileNetV4-YOLOv8m achieves competitive detection accuracy among the compared FP32 models, with a representative $mAP_{50}$ of 93.1% and $mAP_{50:95}$ of 58.9%. Compared with the original YOLOv8m baseline, the proposed model increases $mAP_{50}$ by 0.6 percentage points and $mAP_{50:95}$ by 0.8 percentage points in the representative run, while reducing the parameter count from 25.9 M to 13.1 M and GFLOPs from 78.9 to 39.6. In addition, the repeated-seed analysis in Table 8 indicates that this performance difference remains stable under different training seeds. Therefore, the advantage of the proposed model should be interpreted primarily as a more favorable accuracy–efficiency trade-off, rather than as a large absolute accuracy improvement.

Compared with recent lightweight and edge-oriented real-time detectors, such as YOLOv8s, YOLOv10s, YOLOv11s, LEAD-YOLO, and YOLO-TS, the proposed model maintains higher detection accuracy with moderate model complexity. For example, YOLOv10s achieves 91.9% $mAP_{50}$ and 57.1% $mAP_{50:95}$ with 8.0 M parameters and 24.5 GFLOPs, while YOLOv11s achieves 91.6% $mAP_{50}$ and 56.9% $mAP_{50:95}$ with 9.4 M parameters and 21.5 GFLOPs. LEAD-YOLO obtains 92.1% $mAP_{50}$ and 57.4% $mAP_{50:95}$ with 7.9 M parameters and 26.1 GFLOPs, showing good lightweight characteristics and real-time performance. YOLO-TS further improves the accuracy to 92.4% $mAP_{50}$ and 57.7% $mAP_{50:95}$ with 10.8 M parameters and 33.5 GFLOPs. Although these lightweight models are more compact or computationally efficient, their detection accuracy is still lower than that of the proposed MobileNetV4-YOLOv8m on the constructed dataset.

YOLOv11m obtains a closer result, with 92.8% $mAP_{50}$ and 58.3% $mAP_{50:95}$, but it requires 20.1 M parameters and 68.0 GFLOPs. In contrast, the proposed MobileNetV4-YOLOv8m achieves higher $mAP_{50}$ and $mAP_{50:95}$ while using fewer parameters and substantially lower computational cost than YOLOv11m. This suggests that simply adopting a compact general-purpose detector may not provide the most suitable balance for vehicle-ejected debris detection, where small and irregular road-surface targets require both efficient feature extraction and effective multi-scale feature adaptation.

The INT8 version of the proposed model further evaluates the feasibility of edge-side deployment. After quantization and deployment on the RDK X5 BPU, MobileNetV4-YOLOv8m-INT8 achieves 112.6 FPS while maintaining 92.2% $mAP_{50}$. Compared with its FP32 counterpart, the quantized model shows only a 0.9-point decrease in $mAP_{50}$, indicating that the proposed architecture remains robust after INT8 quantization. Since the FP32 and INT8 FPS values are measured on different hardware platforms, the INT8 result is not used for direct speed ranking with the workstation-based FP32 models, but is reported separately as the deployment performance of the proposed model on the target RDK X5 BPU. Therefore, this result mainly demonstrates the real-time feasibility of the proposed framework on the target edge accelerator, rather than serving as a complete same-hardware INT8 benchmark against all baseline detectors.

Overall, although the comparison does not exhaust all possible object detection frameworks, it covers representative accuracy-oriented, lightweight, and real-time edge-oriented detectors under a unified dataset and training protocol. The comparison indicates that the proposed MobileNetV4-YOLOv8m framework is not merely a smaller version of YOLOv8m. By combining a MobileNetV4 backbone, scale-specific channel alignment, and INT8-oriented edge deployment, the proposed framework provides a practical accuracy–efficiency trade-off for vehicle-ejected debris detection under vehicle-mounted edge-computing constraints.

### 3.6. Visualization Analysis

To further evaluate the practical detection behavior of the proposed model, qualitative visualization results are provided in this section. The selected samples are taken from the test set and cover three representative scenarios: near-range debris detection, distant small-object detection, and nighttime reflective-interference detection. For each scenario, the prediction result of the YOLOv8m baseline is shown on the left, while the result of the proposed MobileNetV4-YOLOv8m model is shown on the right.

**Figure 7:**
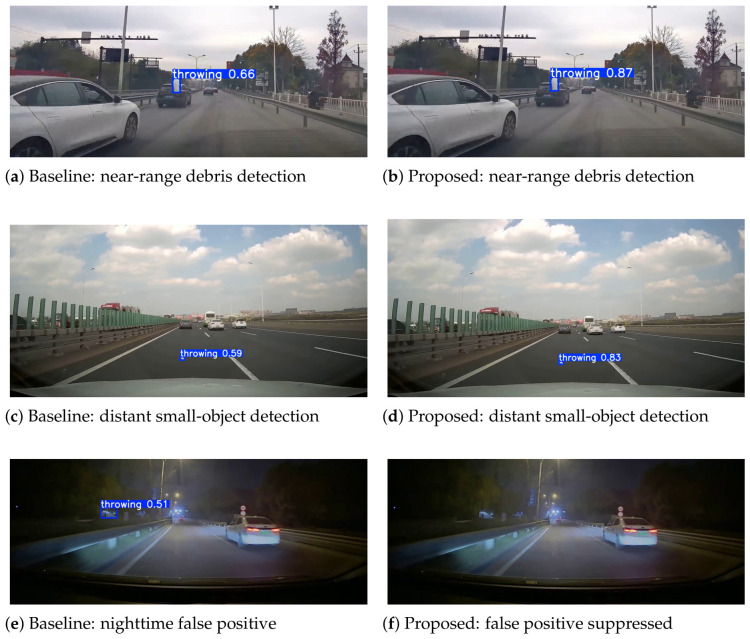
Qualitative comparison between the YOLOv8m baseline and the proposed MobileNetV4-YOLOv8m model. The first row shows near-range debris detection, the second row shows distant small-object detection, and the third row shows a nighttime reflective-interference case where the baseline produces a false positive while the proposed model suppresses it.

As shown in Figure 7, the proposed model maintains stable detection performance under different target distances and illumination conditions. In the near-range debris detection case, both models can localize the target, which indicates that replacing the original backbone with MobileNetV4 does not weaken the basic detection capability of YOLOv8m. In the distant small-object case, the target occupies only a limited region of the road surface, while the proposed model produces a higher-confidence prediction than the baseline. This suggests that the channel alignment module helps preserve useful multi-scale features for small debris detection.

In the nighttime reflective-interference case, the baseline model generates a false positive due to strong reflection and low-light background interference. In contrast, the proposed model suppresses this false detection, showing better robustness under challenging illumination conditions. These qualitative results are consistent with the quantitative results in the ablation study, demonstrating that the proposed MobileNetV4-YOLOv8m model improves detection stability while reducing model complexity.

Several failure modes remain under challenging vehicle-mounted sensing conditions. First, missed detections may occur when debris objects are extremely small, especially when the target occupies only a few pixels after resizing to 640×640. Second, low-contrast scenes may reduce the separability between debris and asphalt, particularly under nighttime illumination, shadowed regions, or dark road surfaces. Third, strong specular reflections on wet asphalt or bright lane markings may form local high-response regions that resemble small debris, leading to occasional false positives. These observations indicate that the proposed model is effective under common vehicle-mounted road scenes, but its robustness can still be affected by extremely small target scale, weak object-background contrast, and reflection-induced hard negatives. Addressing these cases will require a larger number of difficult samples and more diverse road-scene data, which will be further investigated in the next stage of this work.

### 3.7. RDK X5 Deployment Results

To verify the practical feasibility of the proposed framework, the INT8-quantized MobileNetV4-YOLOv8m model was deployed on the RDK X5 edge computing platform for real-time vehicle-mounted inference. A 2-megapixel high-definition camera was used as the visual input device to capture road-scene video frames. The captured frames were resized to 640 × 640 and then fed into the BPU for debris-ejection detection. The deployment pipeline included camera acquisition, image pre-processing, BPU inference, post-processing with non-maximum suppression (NMS), and result visualization.

As shown in Table 13, the proposed INT8 model achieves an average inference speed of 112.6 FPS on the RDK X5 BPU, corresponding to an average BPU inference latency of approximately 8.9 ms per frame. This result indicates that the proposed model satisfies the real-time requirement of vehicle-mounted edge perception. Compared with the FP32 model evaluated on the training workstation, the INT8 model further improves inference throughput while maintaining a limited accuracy degradation, as reported in Table 7. Therefore, the proposed model is not only lightweight in terms of parameters and computational cost, but also practically deployable on an embedded edge accelerator.

In addition to quantitative runtime evaluation, real-world recognition results were collected under different illumination conditions, including daytime, dusk low-light, and nighttime scenarios. Unlike the offline comparison in Figure 7, this experiment focuses on the recognition results generated directly by the edge-side platform during real-time inference.

**Figure 8:**
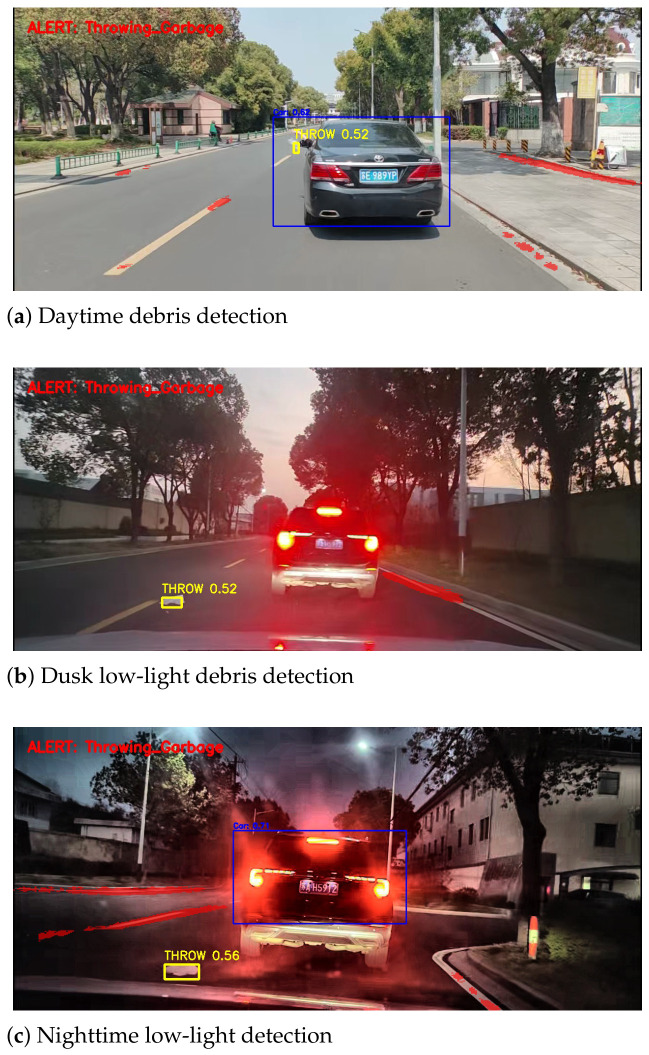
Recognition results of debris-ejection cases on the RDK X5 platform under different illumination conditions. The proposed MobileNetV4-YOLOv8m-INT8 model detects debris in daytime, dusk low-light, and nighttime reflective-interference scenarios. The nighttime frame is affected by real-time low-light image enhancement or camera-side exposure compensation during vehicle-mounted inference.

As shown in Figure 8, the deployed INT8 model can recognize debris-ejection cases from video frames captured by the 2-megapixel high-definition camera under different illumination conditions. In the daytime scenario, the target has relatively clear boundaries and sufficient contrast against the road surface, allowing the model to localize the debris accurately. In the dusk low-light scenario, the model still detects the target despite reduced illumination and weaker object-road contrast. In the nighttime scenario, the image quality is affected by insufficient illumination, strong vehicle-light reflection, and real-time image enhancement or camera-side exposure compensation. Although these factors introduce slight blur and noise, the deployed model still produces a correct detection result.

It should be noted that the nighttime result reflects a practical challenge in vehicle-mounted deployment rather than an offline dataset artifact. In real road environments, camera exposure adjustment, headlight reflection, and low-light enhancement may change the visual appearance of small road-surface objects. The successful detection under this condition suggests that the proposed MobileNetV4-YOLOv8m-INT8 model maintains practical robustness under challenging illumination.

Overall, the RDK X5 deployment experiment demonstrates not only the computational feasibility of the proposed INT8 model, but also its applicability to real-time vehicle-mounted traffic sensing. Combined with the quantitative results in Table 13 and the ablation results in Table 7, the edge-side recognition results further verify the practical value of the proposed lightweight detection framework.

## 4. Conclusions

This paper proposed a lightweight detection framework for vehicle-ejected debris on edge computing platforms. The CSPDarknet backbone of YOLOv8m was replaced by MobileNetV4-conv-medium, and a Channel Alignment Module (CAM) was introduced to bridge the dimensional mismatch between the new backbone and the original PANet neck. The CAM also serves as a learnable projection that reshapes backbone features to fit the downstream representation, which helps recover the accuracy lost from direct backbone replacement. The full model was then quantized to INT8 and deployed on the RDK X5 BPU through the Horizon OpenExplorer pipeline.

An experimental dataset containing 4328 annotated and augmented image samples was prepared from real vehicle-mounted road videos for model training and evaluation, covering urban and suburban traffic, day and night illumination, and a range of debris categories. 67% of the annotated objects fall in the small-object range ($area<32^{2}$ pixels), confirming that this task is dominated by small targets under typical traffic camera geometry. On this dataset, the proposed model reaches 93.1% $mAP_{50}$ and 58.9% $mAP_{50:95}$, while reducing parameters from 25.9 M to 13.1 M (a 49.4% decrease) and GFLOPs from 78.9 to 39.6 (a 49.8% decrease) relative to the YOLOv8m baseline. Inference speed on the laptop GPU improves from 42.3 to 69.8 FPS, and after INT8 quantization the deployed model runs at 112.6 FPS on the RDK X5 BPU at 5W power, with the $mAP_{50}$ dropping by only 0.9 points to 92.2%. These results indicate that the proposed framework can deliver both detection accuracy and real-time efficiency suitable for in-vehicle traffic enforcement applications.

However, several limitations remain. The dataset was collected in a limited geographical region and under restricted weather conditions; performance under heavy rain, fog, or snow has not been verified. Detection on extremely small targets (below 8×8 pixels) and on objects whose color closely matches the road surface is still unreliable. The model is also restricted to a single-class formulation, which is sufficient for violation event recognition but does not distinguish between specific debris categories.

Although a complete event-level error analysis remains future work, the observed false-positive and false-negative cases reveal several typical failure patterns. False positives mainly occur when visually similar road-surface interference, such as lane-marking fragments, fallen leaves, wet asphalt reflections, tire marks, and shadow boundaries, exhibits texture or shape characteristics similar to small debris objects. False negatives mainly occur when debris targets are extremely small, have low contrast with the road surface, are partially occluded, or appear near image boundaries. These cases indicate that the proposed framework is effective under common vehicle-mounted road scenes, but its robustness is still limited under extremely small-target, low-contrast, and strong-reflection conditions.

Future work will focus on incorporating temporal cues across consecutive frames to exploit the motion patterns of ejected debris, expanding the dataset to adverse weather, reflective road surfaces, long-distance small-object cases, and broader geographical coverage, and exploring quantization-aware training in place of the current post-training quantization. In addition, event-level ejection verification, offending-vehicle attribution, temporal consistency modeling, false-accusation risk control, and same-hardware INT8 deployment comparisons with representative baselines such as YOLOv8m-INT8 and YOLOv11m-INT8 on the RDK X5 BPU will be further investigated.

## Figures and Tables

**Table 1:** Stage configuration of MobileNetV4-conv-medium.

Stage	Output Resolution	Output Channels	Stride
Stem (Conv 3×3)	320×320	32	2
Stage 1	160×160	48	2
Stage 2 (→P3)	80×80	80	2
Stage 3 (→P4)	40×40	160	2
Stage 4 (→P5)	20×20	960	2

**Table 2:** Summary of data acquisition and annotation settings.

Item	Description
Source videos	42 vehicle-mounted road videos
Total acquisition duration	Approximately 90 min
Camera view	Front-view vehicle-mounted camera
Video resolution/frame rate	1920 × 1080, 30 FPS
Road scenarios	Urban roads, suburban roads, intersections, and low-light scenes
Annotation format	YOLO bounding-box format
Target category	Single class: throwing debris
Final image samples	4328 annotated images
Dataset split	Training/validation/test = 7:2:1

**Table 3:** Dataset partition for vehicle-ejected debris detection.

Subset	Image Samples	Proportion
Training set	3030	70.0%
Validation set	866	20.0%
Test set	432	10.0%
Total	4328	100%

**Table 4:** Object scale distribution in the constructed dataset.

Object Scale	Instances	Proportion
Small ($area<32^{2}$)	2899	67%
Medium ($32^{2}\leq area<96^{2}$)	1342	31%
Large ($area\geq 96^{2}$)	87	2%

**Table 5:** Hardware and software configuration.

Item	Configuration
Operating system	Windows 11
CPU	13th Gen Intel Core i9
GPU	NVIDIA GeForce RTX 4080 Laptop
GPU memory	12 GB
System memory	32 GB
Deep learning framework	PyTorch 2.x
CUDA version	11.8
Python version	3.10
YOLO framework	Ultralytics YOLOv8
Training resolution	640×640
Edge platform	RDK X5
Camera	2-megapixel high-definition camera
BPU computing power	10 TOPS INT8
Deployment toolkit	Horizon OpenExplorer (Horizon Robotics, Beijing, China, Ubuntu 20.04, X5 GPU, v1.2.8)

**Table 6:** Training hyperparameter settings.

Hyperparameter	Value
Input size	640×640
Epochs	200
Batch size	16
Optimizer	SGD
Initial learning rate	0.01
Weight decay	0.0005
Momentum	0.937
Warm-up epochs	3
Data augmentation	Mosaic, HSV, scaling, flipping
Loss weights	box: 7.5, cls: 0.5, dfl: 1.5

**Table 7:** Ablation study on the proposed components.

Model	MobileNetV4	CAM	P (%)	R (%)	${\mathrm{mAP}}_{50}$ (%)	${\mathrm{mAP}}_{50:95}$ (%)	Params (M)	FPS
YOLOv8m baseline	–	–	91.2	87.4	92.5	58.1	25.9	42.3
MobileNetV4 backbone + minimal neck-channel adjustment (no CAM)	√	–	88.9	85.2	90.1	55.2	12.8	72.5
MobileNetV4 backbone + CAM	√	√	91.8	88.1	93.1	58.9	13.1	69.8
INT8 deployment (BPU)	√	√	91.0	87.3	92.2	57.4	13.1	112.6

Note: “√” indicates that the corresponding component is used in the model configuration.

**Table 8:** Repeated-run comparison under five different random seeds. Results are reported as mean ± standard deviation.

Model	P (%)	R (%)	${\mathrm{mAP}}_{50}$ (%)	95% CI of ${\mathrm{mAP}}_{50}$	${\mathrm{mAP}}_{50:95}$ (%)	Params (M)	GFLOPs
YOLOv8m baseline	91.20±0.16	87.40±0.16	92.50±0.16	[92.30,92.70]	58.10±0.16	25.9	78.9
MobileNetV4-YOLOv8m	91.84±0.11	88.10±0.16	93.08±0.15	[92.90,93.26]	58.88±0.15	13.1	39.6

**Table 9:** Ablation study on different channel adaptation strategies.

Channel Adaptation Strategy	P (%)	R (%)	${\mathrm{mAP}}_{50}$ (%)	${\mathrm{mAP}}_{50:95}$ (%)	Params (M)	GFLOPs	FPS
Minimal neck-channel adjustment	88.9	85.2	90.1	55.2	12.8	38.7	72.5
Channel padding/cropping	89.4	85.7	90.7	55.8	12.8	38.8	72.1
Plain 1×1 Conv	90.7	86.6	91.8	57.1	12.9	39.1	71.4
Proposed CAM	91.8	88.1	93.1	58.9	13.1	39.6	69.8

**Table 10:** Internal ablation study of the proposed CAM.

CAM Variant	P (%)	R (%)	${\mathrm{mAP}}_{50}$ (%)	${\mathrm{mAP}}_{50:95}$ (%)	Params (M)	GFLOPs	FPS
Plain 1×1 Conv	90.7	86.6	91.8	57.1	12.9	39.1	71.4
1×1 Conv + BN	91.1	87.0	92.2	57.6	13.0	39.2	70.9
1×1 Conv + BN + SiLU	91.4	87.4	92.6	58.0	13.0	39.3	70.5
Non-scale-specific mapping	91.3	87.2	92.4	57.8	13.0	39.2	70.7
P3 alignment only	91.2	87.1	92.5	57.9	13.0	39.3	70.4
P4 alignment only	90.9	86.8	92.0	57.4	12.9	39.2	70.8
P5 alignment only	90.8	86.7	91.9	57.3	12.9	39.2	70.9
Full scale-specific CAM	91.8	88.1	93.1	58.9	13.1	39.6	69.8

**Table 11:** Comparison of different lightweight backbones under the same YOLOv8m neck and head.

Backbone	P (%)	R (%)	${\mathrm{mAP}}_{50}$ (%)	${\mathrm{mAP}}_{50:95}$ (%)	Params (M)	GFLOPs	FPS
CSPDarknet baseline	91.2	87.4	92.5	58.1	25.9	78.9	42.3
ShuffleNetV2 + CAM	88.8	85.3	90.6	55.7	8.7	24.9	86.3
GhostNet + CAM	89.9	86.1	91.5	56.4	10.4	29.7	80.6
MobileNetV3-Large + CAM	90.8	86.9	92.1	57.5	12.3	34.8	72.8
MobileNetV4-conv-medium + CAM	91.8	88.1	93.1	58.9	13.1	39.6	69.8

**Table 12:** Comparison with representative object detection models.

Model	P (%)	R (%)	${\mathrm{mAP}}_{50}$ (%)	${\mathrm{mAP}}_{50:95}$ (%)	Params (M)	GFLOPs	FPS
Faster R-CNN	88.6	84.2	90.3	55.7	41.2	134.5	18.6
YOLOv8n	86.7	82.4	88.1	52.6	3.2	8.7	96.4
YOLOv8s	89.8	85.9	91.2	56.4	11.2	28.6	68.5
YOLOv8m	91.2	87.4	92.5	58.1	25.9	78.9	42.3
YOLOv10s	90.4	86.6	91.9	57.1	8.0	24.5	73.4
YOLOv11s	90.1	86.3	91.6	56.9	9.4	21.5	71.2
YOLOv11m	91.5	87.7	92.8	58.3	20.1	68.0	48.7
LEAD-YOLO	90.7	86.9	92.1	57.4	7.9	26.1	74.2
YOLO-TS	91.1	87.2	92.4	57.7	10.8	33.5	65.9
MobileNetV4-YOLOv8m	91.8	88.1	93.1	58.9	13.1	39.6	69.8
MobileNetV4-YOLOv8m-INT8	91.0	87.3	92.2	57.4	13.1	39.6	112.6

Note: The FPS values of FP32 models were measured on the training workstation. The FPS of MobileNetV4-YOLOv8m-INT8 was measured on the RDK X5 BPU and is reported only to indicate edge-deployment performance, rather than for direct cross-platform speed comparison.

**Table 13:** Runtime performance of the proposed INT8 model on the RDK X5 platform.

Metric	Value
Input camera	2-megapixel HD camera
Input resolution	640 × 640
Model precision	INT8
BPU inference latency	8.9 ms
Average inference speed	112.6 FPS
Pre-processing	Image resizing and normalization
Post-processing	NMS and result rendering
Deployment toolkit	Horizon OpenExplorer
Edge accelerator	RDK X5 BPU, 10 TOPS INT8

## Data Availability

The original contributions presented in this study are included in the article. Further inquiries can be directed to the corresponding author.
